# Characteristics and predictors of home injury hazards among toddlers in Wenzhou, China: a community-based cross-sectional study

**DOI:** 10.1186/1471-2458-14-638

**Published:** 2014-06-23

**Authors:** Xianyun Qiu, Chintana Wacharasin, Wannee Deoisres, Jifang Yu, Qiong Zheng

**Affiliations:** 1 School of Nursing, Wenzhou Medical University, University-town, Wenzhou, Zhejiang, P.R. China; 2 Nursing Faculty, Burapha University, Bangsaen Road, Chonburi, Thailand; 3 ICU, The First Affiliated Hospital of Wenzhou Medical University, Ouhai district, Wenzhou, Zhejiang, P.R. China

**Keywords:** Home hazards, Unintentional home injuries, Children, Parent, Family

## Abstract

**Background:**

Home hazards are associated with toddlers receiving unintentional home injuries (UHI). These result in not only physical and psychological difficulties for children, but also economic losses and additional stress for their families. Few researchers pay attention to predictors of home hazards among toddlers in a systematic way. The purpose of this study is firstly to describe the characteristics of homes with hazards and secondly to explore the predicted relationship of children, parents and family factors to home hazards among toddlers aged 24–47 months in Wenzhou, China.

**Methods:**

A random cluster sampling was employed to select 366 parents having children aged 24 – 47 months from 13 kindergartens between March and April of 2012. Four instruments assessed home hazards, demographics, parent’s awareness of UHI, as well as family functioning.

**Results:**

Descriptive statistics showed that the mean of home hazards was 12.29 (SD = 6.39). The nine kinds of home hazards that were identified in over 50% of households were: plastic bags (74.3%), coin buttons (69.1%), and toys with small components (66.7%) etc. Multivariate linear regression revealed that the predictors of home hazards were the child’s age, the child’s residential status and family functioning (b = .19, 2.02, - .07, p < .01, < .05 and < .01, respectively).

**Conclusions:**

The results showed that a higher number of home hazards were significantly attributed to older toddlers, migrant toddlers and poorer family functioning. This result suggested that heath care providers should focus on the vulnerable family and help the parents assess home hazards. Further study is needed to find interventions on how to manage home hazards for toddlers in China.

## Background

Unintentional injury is the leading cause of morbidity and mortality among children under five years globally [1,2]. Furthermore, 1/3 to over 1/2 of injuries occur at home [3,4]. Unintentional home injuries (UHI) result in not only substantial health problems to children and negative impacts on their families, but also in large financial losses for countries [5-8]. One key factor affecting UHI is the physical environment in the home, and is mainly composed of situations where there is a high potential for a variety of injury hazards. A number of research studies indicated that home hazards were a risk factor for home injuries among young children [9-15].

Theoretically, home hazards are more likely to be influenced by factors in three domains: child factors, parent factors and family factors. For child factors, age and/or gender have a possible impact on home hazards. For example, boys tend to be more boisterous and mischievous than girls, which could increase their exposure to home hazards [16]. Parent factors also need to be considered since parents serve as home designers and managers. A few research findings found that parents’ low educational status and low awareness of UHI was positively associated with home hazards [17-20]. Lastly, the family is one of the social environments in which home hazards exist. Family factors have an impact on home hazards. These factors include family demographics (i.e. number of family, family income and housing tenancy) and family functioning. In general, families with more members, low income or poor housing would be positively associated with more home hazards. However, these alternative explanations have not been adequately tested.

An increasing number of studies have paid attention to occurrence and causal etiology of unintentional injuries among children, however, few attempts have focused on home hazards and related factors [10-14,17-20]. The objectives of the present study are to (1) describe the characteristics of home hazards for young children aged 24–47 months in Wenzhou, China; and (2) examine the predicted relationship between child factors, parent factors, family factors, and home hazards.

In Wenzhou city, the population of children 24-47 months is approaching 76 thousand persons [21]. The incidence rate of UHI to this aggregate (40.4%) is quite high and home hazards are a key contributing factor [6]; therefore this study would be beneficial to assist in the control of home hazards and the prevention of UHI in Wenzhou.

## Methods

### Population and sampling

This is community-based cross-sectional study. The population of interest was parents having children within the age range of 24–47 months residing in Wenzhou City for more than 6 months. The parents were also able to read and understand Chinese. Given hierarchical linear regression, the ratio of cases-to-independent variables (IV) is no less than 20:1 [22]. Because of 15 independent variables, at least three hundred samples were needed.

A random cluster sampling was performed to select one and two communities from the nine suburban and twelve urban communities, respectively, and then select 13 kindergartens. All kindergartens which enrolled toddlers aged 24–47 months were selected except for two; those leaders would not permit the survey. Finally, 28 classes were selected to collect data. Samples were recruited from the parents who had their 24–47 months old children attending 28 classes from 13 kindergartens.

### Research instruments

#### ***There were four instruments***

The Assessment Tool of Domestic Hazards (ATDH) (see Additional file 1) was used to evaluate the number of home injury hazards [20]. Home hazards are operationally defined as an agent stored in a home environment that has the potential of a harmful feature that could lead to UHI [11]. ATDH was created by Japanese researchers and modified by Chinese researchers [20,23]. The content validity of the Chinese version was confirmed by qualitative analysis and three experts. Its internal consistency was .85 and test- retest reliability was .69. The tool includes 37 items. Every item explained one hazard. The 37 injury hazards were related to six common UHI in China [24]: suffocation (e.g., saran wrap, peanut or bean, etc.), poisoning (e.g., medicine, pesticide or detergent etc.), sharp instrument injury (e.g., sharp toy, knife etc.), burns (e.g., hot tea, hot kettle etc.), electric shock (e.g., uncovered electric socket lower than 1 meter), and fall (e.g., wet floor). Every item asked the participants whether their children had the ability to reach for or run with one of the listed hazards. Each item had two answer choices: “yes” (1 point) or “No” (0 point). The total score was 37 points.

A demographic questionnaire was utilized to assess the characteristics of the children (i.e. age, gender, birth order, residence), parents (i.e. type, education, occupation), and family (i.e. family type, number of family numbers, family income and house type). In this study, migrant children are defined as temporary residents in a city for more than six months, while local children were categorized as permanent residents in the city [25].

Situation Awareness of Toddlers’ Parents to Domestic Injuries (SATPDI) was performed to assess parental awareness of UHI [26]. It contains 24 questions which asks parents to identify home hazards from three photos presenting a simulative dining room, sitting room and bedroom, and to choose dangerous conditions to their own children and to choose the consequence of the actions. Crobach’s alpha was .88 and test-retest reliability was .79.

The Family Adaptability and Cohesion Evaluation Scale, the 2nd edition-Chinese version (FACESII-CV) was used to measure family functioning [27]. Family functioning was operationally defined as the degree of family members’ help and support provided to each other and the ability of the family to solve problems. There were 28 items using a likert-type scale. The Cronbach’s alpha was .80.

### Data collection

Six trained research assistants met subjects when they went to pick up their children at kindergartens. The research team explained to eligible parents the research objectives, the advantages and disadvantages for participating in the research, as well as their right to withdraw at any time. After informed consent was obtained, one envelope with the questionnaire and instructions was handed to every parent. The instructions told them how to fill in the questionnaire and how to contact the researcher. After 1 to 2 days, parents brought the completed questionnaires back to the class and the research assistants reviewed them for completeness. A small gift was given to every parent as an acknowledgement of his or her participation. Overall, 366 completed questionnaires from 491 eligible subjects. The response rate was 74.5%. The majority of non-responders were not interested in the project or too busy; some were unwilling to complete questionnaires due to the length of the questionnaires or private matters such as family income and birth order requests.

### Data analysis

SPSS13.0 was used for statistical analyses. Hierarchical linear regression was employed to determine factors for home hazards. The alpha level for significance was set at .05. Two of the variables; parent’s age and family income were not normally distributed so were transformed into ordinal variables. The intercorrelations between all independent variables were not significant (less than .75) [22]. Pearson product moment correlation coefficients (r) were performed to examine bivariate correlations between child factors, parent factors, family factors and home hazards. Finally, all significant independent variables were put into a hierarchical model.

## Results

### Demographic characteristics

The sample consisted of 366 parents who had toddlers ranging from 24 to 47 months old. The mean age of their children was 36.9 months. They included 191 boys and 175 girls. Among them, the number of local families 218 (59.6%) was higher than migrants (40.4%). Other social demographic data are presented in Table 1.

**Table 1:** Demographic characteristics of children, parents and families (N = 366)

**Variables**	**n(%)**	**Average, SD, range**
**Child’s**		
Age (months)		Mean = 36.9 SD = 4**.**6, range: 24 - 47
24 – 35	115(31.4%)	
36 – 47	251(68.6%)	
Gender		
Male	191(52.2%)	
Female	175(47.8%)	
Resident status		
Local	218(59.6%)	
Migrant	148(40.4%)	
Birth order		
First	276(75.4%)	
Second or third	90(24.6%)	
**Parent’s** type		
Type		
Mother	267(73.0%)	
Others	99(27.0%)	
Age (years)		Median = 31, range: 20 -69
≤31	197(53.8%)	
>31	169(46.2%)	
Education		Mean = 11.7, SD = 2.9, range: 5 - 18
High school or lower	200(54.6%)	
Diploma or higher	166(45.4%)	
Occupation		
Housewife	71(19.4%)	
Employed	295(80.6%)	
**Family**		
Community		
Urban	280(76.5%)	
Suburban	86(23.5%)	
Family type		
Nuclear	200(54.6%)	
Extended	166(45.4%)	
No. of family members		Mean = 4.2, SD = 1.2, range: 3 - 8
4 or less	221(60.3%)	
5 or more	145(39.7%)	
Family income (Chinese Yuan per month)		Median = 10000, Range: 2000 – 450000
≤10000	254(69.4%)	
>10000	112(30.6%)	
House type		
Owned	212(57.9%)	
Other’s	154(42.1%)	

### Description of specific home hazards

**Figure 1:**
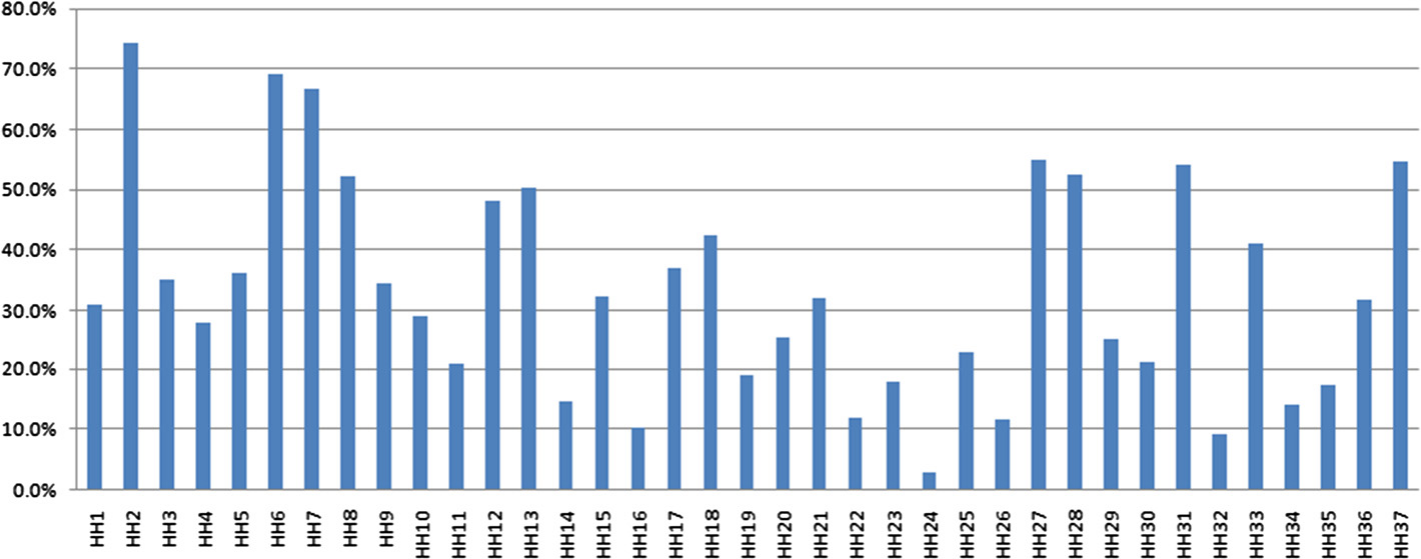
**Distribution of households with every reported hazard for all households.** Note: children can reach the following home hazards (HH1 ~ HH32). HH1: food wrap; HH2: plastic bag. HH3 string; HH4: long band; HH5: peanuts or beans; HH6: coin or button; HH7: small detachable toy; HH8: sunflower seeds; HH9: cigarettes or ashtray; HH10: medicine; HH11: pesticide or detergent; HH12: shampoo or body lotion; HH13: make-up; HH14: knives; HH15: scissors; HH16: pins; HH17: sharp toys; HH18: sharp table; HH19: hot tea or soup; HH20: thermos bottle or water dispenser; HH21: rice cooker; HH22: hot frying pans; HH23: hot kettle; HH24: electric irons; HH25: heaters; HH26: hot-water taps; HH27: low socket; HH28: socket without cover; HH29: no guardrails in window or balcony; HH30: stool by window; HH31: stool by table or cabinet; HH32: shipping floor. Children would run with HH33 ~ HH37, HH37: chopstick or spoon; HH34: tally; HH35: toothpick; HH36: toothbrush; HH37: rules or pencils.

On average, 12 injury hazards were reported for all households (SD = 6.39 range 0–36). The percentage of households with hazards fell between 2.7% and 74.3% (Figure 1). Electric irons and slipping on the floor were reported at 2.7% and 9% respectively in the sample, whereas more than 50% of families reported 9 additional hazards: plastic bags (74.3%), coins/buttons (69.1%), toys with small components (66.7%), low sockets (54.9%), sharp rulers or pencils (54.6%), stools by a table or cabinet (54.1%), uncovered sockets (52.5%), sunflower seeds (52.2%) and make-up (50.3%).

### Relationship between independent variables and home injury hazards

Child’s age, child’s resident status, child’s birth order, community, house type were positively correlated with home hazards (r = .15, .23, .11, .14, .1, and .23; p < .01, 01, 05, 01, 05, and .01, respectively). Parents’ education and family functioning were negatively correlated with home hazards (r = -.22 and -.19; p < .01, and .01 respectively) (Table 2).

**Table 2:** Correlation of home hazards by independent variables (N = 366)

**Variables**	**Home hazards**	
	**r**	**P**
Child’s age	.154**	.003
Parent’s education	-.222***	.000
Parent’s awareness	-.076	.144
No. of family members	.104*	.047
Family function	-.192***	.000
	**r**_**s**_	**P**
**Child’s**		
Gender(female)^a^	-.049	.354
Resident status(migrant)^b^	.232***	.000
Birth order(second or third)^c^	.114*	.029
**Parent’s**		
Type(others)^d^	.042	.421
Age (>31)^e^	.086	.100
Occupation(employed)^f^	.088	.118
**Family**		
Community(suburban)^g^	.144**	.006
Family type(extended)^h^	.069	.187
Family income(>10000)^i^	.056	.289
House type(other’s)^j^	.225***	.000

Note: *p < .05, **p < .01, ***p < .001 r is Pearson’s correlation coefficient, r_s_ is Spearman’s correlation coefficient.

^a^reference category is ‘male’; ^b^reference category is ‘local’; ^c^reference category is ‘first’; ^d^reference category is ‘mother’; ^e^reference category is ‘≦31’; ^f^reference category is ‘housewife’; ^g^reference category is ‘urban’; ^h^reference category is ‘nuclear’; ^i^reference category is ‘≤10000’; ^j^reference category is ‘own’.

### Predictors of home hazards

Sets of the eight previously discussed significant variables were entered hierarchically into a regression model to determine their relative importance in the prediction of home hazards (Table 3). The Child’s age, child’s resident status and family functioning were predictors of exposure to home hazards. Older toddlers were exposed to more injury hazards in their home (b = .19, p < .01). Lower family functioning resulted in more home hazards (b = -.07, p < .01). Migrant children were shown to be exposed to more home hazards (b = 2.02, p < .05) compared with local children. The three predictors accounted for 12.9% of the variance of home hazards, with nearly three-fourths of the explained variance (9.1%) explained by child factors.

**Table 3:** Predictors of home hazards by hierarchical linear regression (N = 366)

**Independent variables**	**Intercept**	**b**	**Beta**	**R**^**2**^	**R**^**2 **^**change**
**Set1**	3.90			.091	.091
Child’s age		.19**	.13	F(2,362) = 12.05	F(3,362) = 12.05
Child’s resident status(migrant)^a^		2.85***	.22		
Child’s birth order(second or third)^b^		1.57*	.11		
**Set 2**	6.78			.097	.007
Child’s age		.19**	.13	F(4.361) = 9.75	F(1,361) = 2.69
Child’s resident status(migrant)^a^		2.19**	.17		
Child’s birth order(second or third)^b^		1.28	.09		
Parent’s education parent		-.22	-.10		
**Set 3**	9.93			.129	.032
Child’s age		.19**	.13	F(8,357) = 6.63	F(4,357) = 3.27
Child’s resident status(migrant)^a^		2.02*	.16		
Child’s birth order(second or third)^b^		.97	.07		
Parent’s education parent		-.06	-.03		
Community(suburban)^c^		.21	.02		
No. of family members		.46	.08		
House type((other’s)^d^		.74	.06		
Family functioning		-.07**	-.16		

Note: *p < .05, **p < .01, ***p < .001.

^a^reference category is ‘local’; ^b^reference category is ‘first’; ^c^reference category is ‘urban’; ^d^reference category is ‘other’s’.

## Discussion

The current study explored the characteristics and predictors of home hazards among children aged 24–47 months (equal to 2–3 years) old in Wenzhou, Zhejiang Province, China. The average percentage of reported injury hazards (33.2%), was consistent with that for toddlers in Anhui province, China (38.5%) [20]; furthermore, 50% or more respondents’ homes contained nine injury hazards, specifically, plastic bags, coins or buttons, toys with small components and sunflower seeds. These were related to suffocation and make-up related to poisoning. Electrical sockets in a low position on the wall and uncovered sockets were related to electrical shock. A stool by a table or cabinet correlated with a fall, and finally, pencils correlated to sharp injuries. These results differ from other findings, which indicated that stoves, open buckets and pedestal fans were the main home hazards in a low-income urban setting of Pakistan [28]. The possible reason for the difference was the dissimilarity of the two countries’ home culture and economics. Overall, the result reveals that homes with the characteristics studied here were more likely to be full of injury hazards for toddlers. It is necessary for children’s healthcare providers to assess the nine home hazards and guide the families to remove home hazards.

The finding revealed that older toddlers were positively correlated with home hazards; in other words, older toddlers had a higher potential for injury from home hazards. The result was similar to an American study that showed that children over 2.5 years old were more susceptible to hazards [17]. A possible explanation could be a wider gap between children’s motor skills and their understanding of dangerous hazards as they developed from 24 to 47 months. Gross motor skills and curiosity exceed cognition as toddlers get older. These two powerful attributes can drive older toddlers to move in a larger area or climb to a higher place than they could when they were younger, thereby older ones would reach more home hazard locations [29]. On the other hand, parents of older toddlers became less vigilant compared with parents of younger ones, despite the fact that those older children were still at risk [30]. This predictor implies that it is important for the children’s health professionals to inform families with older toddlers, especially those in the age group of 36 months or older about the linkage between children’s age and home hazards, and to educate them to assess their homes for hazards and to take steps to eliminate the hazards. Future research should be aimed at developing interventions to reduce home hazards or prevent children’s access to home hazards.

Additionally, the findings show that migrant children are exposed to more home hazards compared to local children. Similarly, one study of toddlers identified that an immigration background was associated with lower use of protective safety measures (jack socket) thus allowing children easy access to injury-related hazards [31]. Several explanations were possible as to why migrant children were exposed to more hazards than local children were. Firstly, migrant children often have extended families, so there are more than three people living in one home. Their families have lower incomes and are likely to live in rental houses that tend to be small, with poor physical environments. Thus, a crowded family and a small house could lead to more home hazard opportunities. Secondly, migrant children have more siblings than local ones, so, their parents may supervise them for shorter time and less closely [30]. The houses of migrant children are characterized as containing a high density of home hazards, meanwhile parental supervision and attention were lower. These are possible explanations to show that the migrant children were more likely to be exposed to home hazards. The result implies that it is important for children’s health professionals to help migrant families with young children remove home hazards and make safe houses.

Interestingly, lower family functioning was a positive predictor of home hazards. Family functioning encompasses family cohesion and family adaptation. Lower family cohesion would bring about more family conflicts, which are more likely to distract parents from the supervisory role in a child’s activity. As a result, their children were more likely to exhibit behavior that makes them more susceptible to home hazards. A family with poorer family adaptation has lower ability to cope with family problems. This kind of family is vulnerable to family stress. High family stress would lead to a parent’s mental distraction and possibly stress or disturb a toddler’s emotional or behavioral utility [32,33]. This parents’ distraction would influence the parent’s awareness of the importance of a safe environment and decrease the ability to cope with home hazards. Children’s behavioral states would embolden the children to touch more home hazards since children have insufficient judgment as to whether the objects are safe or not. This point also reveals that one way to decrease home hazards is to improve family functioning.

### Limitations and strengths

Some limitations were seen in this study. Samples were recruited only from the parents whose children attended kindergartens. The same age children who did not attend kindergartens were excluded. This might affect the prevalence of home hazards. Furthermore, data collection might produce bias in the data since the subjects filled out the questionnaires at home. A few parents might finish the task with other family members and not by themselves. This would influence the validity of the study. Despite these limitations, characteristics and predictors of home hazards were systematically analyzed among children aged 24–47 months old in Wenzhou of China.

## Conclusions

A large group of hazards existed in the home where toddlers live in Wenzhou, China, and nine hazards are especially prominent: plastic bags, coins or buttons, toys with small components and sunflower seeds, make-up, lower sockets, uncovered sockets, stools by a table or cabinet and sharp rulers or pencils. The study found that the risk factors for home hazards were older toddlers, migrant children and lower family functioning. Health professionals should pay more attention to vulnerable families with these risk factors and educate them to assess home hazards and manage or store them in safe places. Further research needs to be conducted to reduce home hazards.

### Ethics approval

The ethics approval was provided by Zhejiang Provincial Education Department, China.

## Competing interests

The authors declare that they have no competing interests.

## Authors’ contributions

All authors meet the requirements for authorship and manuscript submission. XQ, CW and WD conceptualized the study design. With the instruction of CW and WD, XQ, JU and QZ conducted to collect and analyze study data. CW and WD also instructed the writing of the manuscript and provided critical review, XQ led to complete all the manuscript sections. All authors read and approved the final manuscript.

## Pre-publication history

The pre-publication history for this paper can be accessed here:

http://www.biomedcentral.com/1471-2458/14/638/prepub

## Supplementary Material

Additional file 1: Assessment tool of home hazards. Click here for file
