# Supplementary material for: Characteristics and predictors of home injury hazards among toddlers in Wenzhou, China: a community-based cross-sectional study
Source: BMC Public Health. 2014 Jun 23;14:638. doi: 10.1186/1471-2458-14-638 (PMC4076497; doi:10.1186/1471-2458-14-638)
Supplement: Additional file 1: — Assessment tool of home hazards. [file 1471-2458-14-638-S1.doc]

**Assessment Tool of Home Hazards**

Instructions: the tool is aimed at knowing about the real hazards of your home. The researchers guarantee not to judge your individual behavior and please fill in the tool easily. The areas which your child **can reach** include floor, table, dressing table, bedstand, cooking bench, washing bench etc. If your answer is “yes”, please circle “1”, answer is “no” and circle “0”.

| No. |  | Yes  (1) | No  (0) |
| --- | --- | --- | --- |
|  | Is there any following item in the areas which your child can reach? |  |  |
| 1 | Saran wrap |  |  |
| 2 | Plastic bag |  |  |
| 3 | String |  |  |
| 4 | Long band |  |  |
| 5 | Peanut or bean |  |  |
| 6 | Coin |  |  |
| 7 | Button |  |  |
| 8 | Melon seeds or sunflower seeds |  |  |
| 9 | Cigarette or ashtray |  |  |
| 10 | Medicine |  |  |
| 11 | Pesticide or detergent |  |  |
| 12 | Shampoo or body lotion |  |  |
| 13 | Makeup |  |  |
| 14 | Knife |  |  |
| 15 | Scissors |  |  |
| 16 | Needle |  |  |
| 17 | toy with sharp corner |  |  |
| 18 | furniture with sharp corner |  |  |
| 19 | Hot tea or hot soup |  |  |
| 20 | Thermos bottle or drinking equipment |  |  |
| 21 | Electric rice cooker |  |  |
| 22 | Hot pot |  |  |
| 23 | Hot kettle |  |  |
| 24 | Electric iron |  |  |
| 25 | Heater |  |  |
| 26 | Switch for hot water |  |  |
| 27 | Is any electric socket higher than 1 meter in your home? |  |  |
| 28 | Is any electric socket covered with protecting box in your home? |  |  |
| 29 | Are there guardrails in the windows or balcony of your home? |  |  |
| 30 | Is there any stepping-stool (e.g., small chairs) beneath the windows at your home? |  |  |
| 31 | Is there any stepping-stool surrounding high furniture such as table or cabinet at your home? |  |  |
| 32 | Is the floor slipping or polishing? |  |  |
|  | Does your baby run anywhere with anything as following? |  |  |
| 33 | Chopsticks or spoon |  |  |
| 34 | Ice cream with tally |  |  |
| 35 | Toothpick |  |  |
| 36 | Toothbrush |  |  |
| 37 | Ruler or pencil |  |  |
